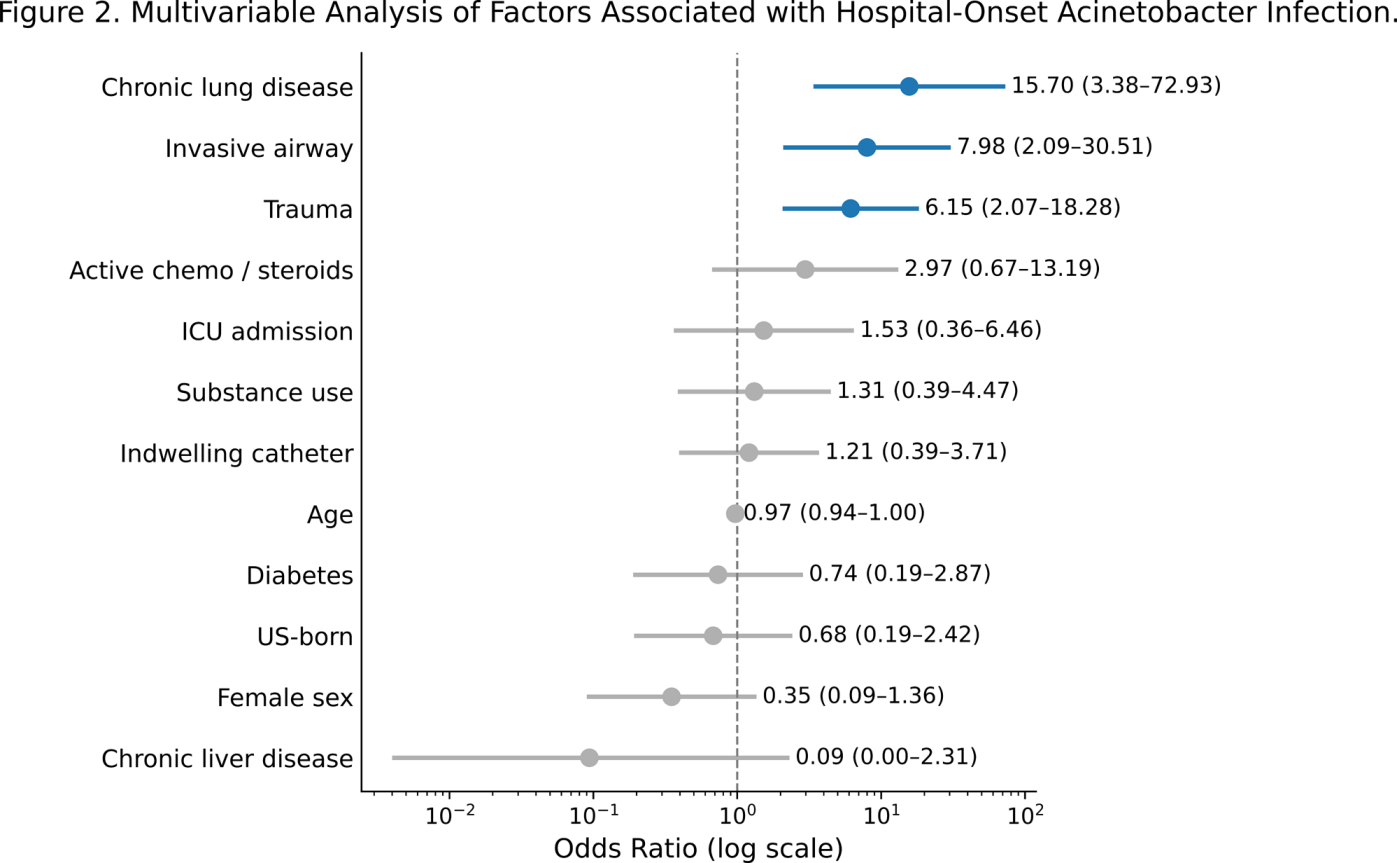# 293 Challenges and Outcomes of Tuberculosis Exposure Investigations Among Healthcare Workers in a Safety-Net Hospital

**DOI:** 10.1017/ash.2026.10651

**Published:** 2026-06-23

**Authors:** Guillermo Rodriguez Nava, Rohana Bruker, Angela Montgomery, Marlee Barton, Jacob McAlinn, Timothy Jenkins, Heather Young

**Affiliations:** 1 Denver Health and Hospital Authority; 2 Denver Health Hospital Authority; 3 Denver Health; 4 Denver Health Medical Center

## Abstract

**Introduction:** Acinetobacter infections are commonly viewed as hospital-onset events linked to critical illness and invasive devices. We evaluated whether this paradigm holds true at our institution by examining a decade of Acinetobacter infections at an urban safety-net trauma center. **Methods:** We conducted a retrospective analysis of patients with Acinetobacter spp. isolated from culture at a 500-bed urban academic safety-net Level I trauma center from January 2015 through December 2025. Clinical and epidemiologic variables were abstracted from the electronic health record. Hospital-onset infection was defined as culture collection on or after hospital day 4 (admission day = day 1). Infections not meeting criteria for hospital onset were classified as community-onset and subsequently assessed for healthcare-associated exposures. Healthcare-associated infection was defined by hospitalization, antibiotic exposure, dialysis, or residence in a skilled nursing facility within 60 days prior to diagnosis. Additional covariates included age, sex, birth place, race/ethnicity, intensive care unit (ICU) admission, active chemotherapy or chronic steroid use, chronic lung disease (e.g., chronic obstructive pulmonary disease or COPD), chronic liver disease (cirrhosis or hepatitis B/C), diabetes, substance use disorder (alcohol or illicit drugs), trauma within 90 days, presence of an invasive airway (endotracheal tube or tracheostomy), indwelling urinary or renal catheters, organism identification (A. baumannii vs non-baumannii species), and final antibiotic used for treatment. To identify factors associated with hospital-onset infection, we used multivariable logistic regression with generalized estimating equations (GEE) to account for within-patient clustering from repeated cultures, reporting adjusted odds ratios (ORs) with 95% confidence intervals (CIs). **Results:** A total of 157 cultures with growth of Acinetobacter spp. were identified during the study period. Acinetobacter baumannii accounted for 69% of isolates. Most cases occurred among inpatients (79%). Hospital-onset infection accounted for 39%, while 61% were community-onset; among community-onset cases, 56% met criteria for healthcare-associated infection. The cohort was predominantly White (45%) or Hispanic (36%). The most common clinical syndromes were ventilator-associated pneumonia (26%), bacteremia (26%), and urinary tract infection (19%). Treatment was administered in 83% of cases, most commonly with levofloxacin (30%) or cefepime (25%). Carbapenem resistance was uncommon (3%). In multivariable GEE analysis accounting for repeated cultures, hospital-onset infection was independently associated with chronic lung disease, recent trauma, and presence of an invasive airway (Figure 2). **Conclusions:** In this safety-net health system, most Acinetobacter infections were community-onset rather than hospital-onset. Hospital-onset infection was strongly associated with markers of critical illness and lung injury rather than demographic factors.

**Figure:**
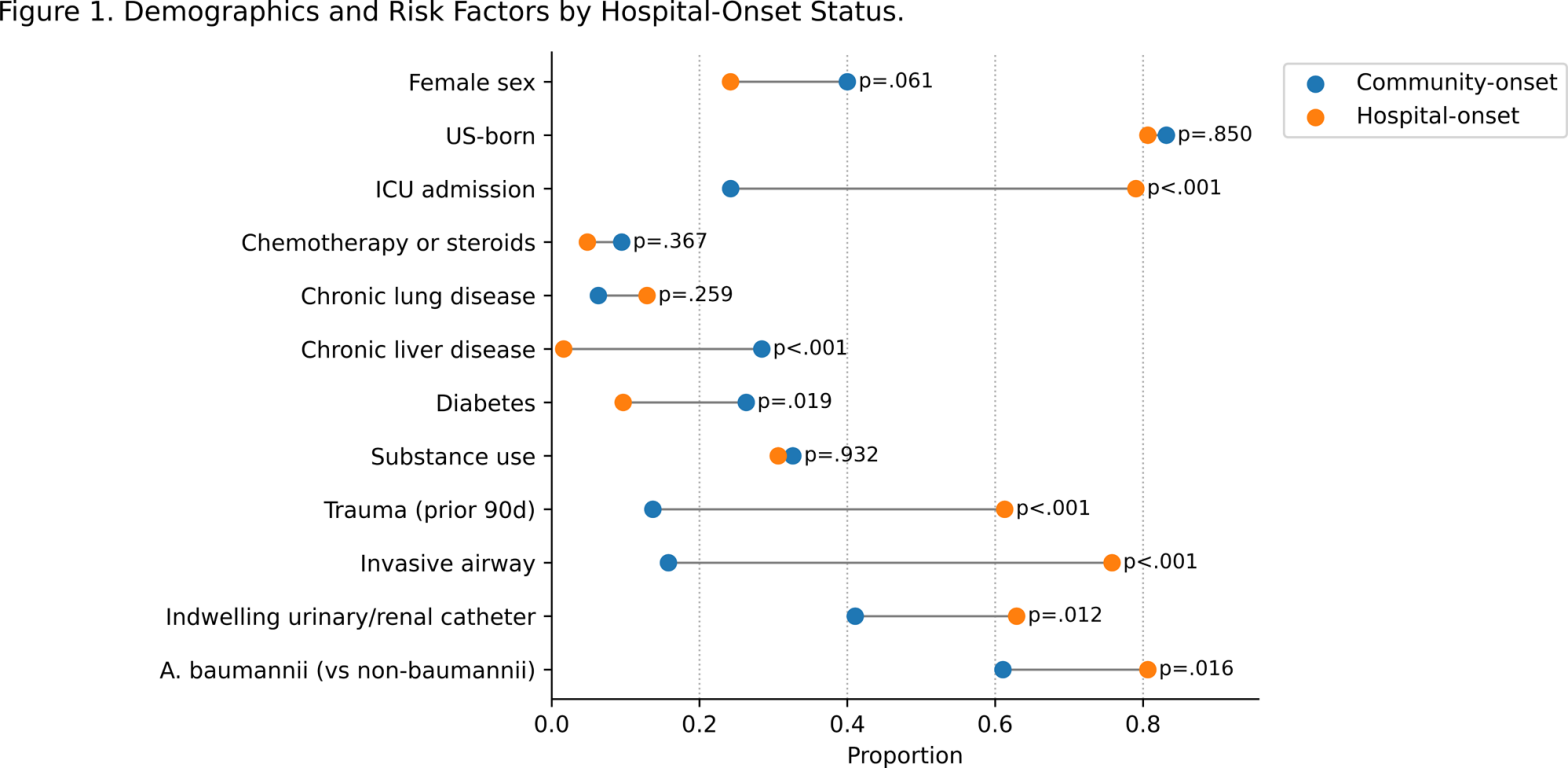

**Figure:**